# New Keys to Early Diagnosis: Muscle Echogenicity, Nerve Ultrasound Patterns, Electrodiagnostic, and Clinical Parameters in 150 Patients with Hereditary Polyneuropathies

**DOI:** 10.1007/s13311-021-01141-3

**Published:** 2021-10-27

**Authors:** Natalie Winter, Debora Vittore, Burkhard Gess, Jörg B. Schulz, Alexander Grimm, Maike F. Dohrn

**Affiliations:** 1 Department of Neurology and Epileptology, Hertie Institute for Clinical Brain Research, University of Tuebingen, Tuebingen, Germany; 2 Department of Neurology, Medical Faculty, RWTH Aachen University, Aachen, Germany; 3 JARA-BRAIN Institute Molecular Neuroscience and Neuroimaging, Jülich Aachen Research Alliance (JARA), FZ Jülich and RWTH University, Jülich, Germany; 4 Department of Human Genetics and John P. Hussman Institute for Human Genomics, Dr. John T. Macdonald Foundation, University of Miami, Miller School of Medicine, Miami, FL USA

**Keywords:** High-resolution nerve ultrasound, Muscle ultrasound, Ultrasound pattern sum score, Entrapment, Charcot-Marie-Tooth disease, Hereditary transthyretin-amyloidosis

## Abstract

Hereditary neuropathies are of variable genotype and phenotype. With upcoming therapies, there is urgent need for early disease recognition and outcome measures. High-resolution nerve and muscle ultrasound is a dynamic, non-invasive, well-established tool in the field of inflammatory and traumatic neuropathies. In this study, we defined nerve and muscle ultrasound parameters as recognition and progression markers in 150 patients with genetically confirmed hereditary neuropathies, including Charcot-Marie-Tooth (CMT) disease (CMT1A, *n* = 55; other CMT1/4, *n* = 28; axonal CMT, *n* = 15; CMTX, *n* = 15), hereditary neuropathy with liability to pressure palsies (HNPP, *n* = 16), hereditary transthyretin-amyloidosis (ATTRv, *n* = 14), and Fabry’s disease (*n* = 7). The CMT1A, followed by the CMT1/4 group, had the most homogeneous enlargement of the nerve cross-sectional areas (CSA) in the ultrasound pattern sum (UPSS) and homogeneity score. Entrapment scores were highest in HNPP, ATTRv amyloidosis, and Fabry’s disease patients. In demyelinating neuropathies, the CSA correlated inversely with nerve conduction studies. The muscle echo intensity was significantly highest in the clinically most affected muscles, which was independent from the underlying disease cause and correlated with muscle strength and disease duration. Further correlations were seen with combined clinical (CMTES-2) and electrophysiological (CMTNS-2) scores of disease severity. We conclude that nerve ultrasound is a helpful tool to distinguish different types of hereditary neuropathies by pattern recognition, whereas muscle ultrasound is an objective parameter for disease severity. The implementation of neuromuscular ultrasound might enrich diagnostic procedures both in clinical routines and research.

**Supplementary Information:**

The online version contains supplementary material available at 10.1007/s13311-021-01141-3.

## Introduction

Hereditary neuropathies are a variable group of progressively disabling diseases, the diagnostic recognition of which is becoming increasingly important because of upcoming treatment approaches.

With an approximate worldwide prevalence of 1 in 2500 individuals [1], Charcot-Marie-Tooth disease (CMT) forms the largest subgroup of hereditary neuropathies. Mutations in more than 80 different genes are known to cause CMT. The typical phenotype encompasses distal symmetric muscle weakness and atrophy, foot deformities such as pes cavus, sensory loss, and reduced or absent tendon reflexes [2, 3]. Corresponding to nerve conduction studies (NCS) and mode of inheritance, a sub-classification into the types CMT 1–4 has been traditionally applied [4, 5]. The most common CMT subtype, CMT1A, is caused by a heterozygous duplication on chromosome 17p12 including the entire *PMP22* gene [6]. A combination of sorbitol, naltrexone, and baclofen is currently in trials for CMT1A [7]. Hereditary transthyretin-related (TTR) amyloidosis is a progressive, systemic disease, that initially manifests with a mixed sensorimotor and autonomic neuropathy [8, 9]. The tetramer-stabilizing small molecule tafamidis [10], as well as the RNA-silencing drugs patisiran [11] and inotersen [12] significantly improve the clinical course, especially when administered early. Another potentially treatable hereditary polyneuropathy is Fabry’s disease, which can manifest with a pure or leading small fiber involvement. Depending on the underlying mutation, approved medications like the chaperone migalastat [13] or an enzyme replacement therapy [14] are available to treat affected mutation carriers, respectively. However, in most types of hereditary neuropathies, therapeutic concepts are still lacking.

For both clinical trials and practice, it is crucial to have valid parameters to identify a specific disease and measure its progression. The traditional neurological examination depends, however, on both the patient’s compliance and the investigator’s experience. Nerve conduction studies (NCS) provide valuable functional information, but are unpleasant for the patient. In cases of advanced axonal damage and loss of nerve stimulability, a classification might no longer be possible. Nerve biopsies might still represent the gold standard to substantiate a certain nerve pathology, but they focus on sensory nerves and cannot be used for follow-up. This fosters the need of another, non-invasive, reproducible, and repeatable method as a representative add-on.

High-resolution nerve ultrasound (HRUS) has recently been introduced to characterize immune-mediated neuropathies [15] and nerve injuries [16]. A diagnostic algorithm for all common subtypes of polyneuropathies is still lacking.

In this study, we examined a large patient cohort (*n* = 150) with genetically confirmed hereditary neuropathies.

Using a standardized protocol [17], we aimed at distinguishing different hereditary neuropathy subtypes. We correlated results with clinical and electrophysiological parameters, suggesting some potential follow-up markers for future clinical trials.

## Methods

### Patient Selection

All patients were either examined at the Neuromuscular Outpatient Clinic of the RWTH Aachen University Hospital, Aachen, Germany, or at the Department of Neurology of the Eberhard Karls University of Tübingen, Tübingen, Germany. The study design conformed to the Declaration of Helsinki, and ethical approval was obtained at both centers. Informed consent was obtained from all participants. All parts of the study protocol were conducted by the same, experienced examiners.

We prospectively examined 113 patients in total, 94 in Aachen and 19 in Tübingen. Retrospective data belonging to another 37 patients previously examined by the same clinicians with the same ultrasound device and examination protocol were additionally included if eligible. For inclusion, patients had to have a hereditary neuropathy with the molecular genetic confirmation of at least one likely pathogenic or pathogenic variant in a known gene associated with the according phenotype. Other, potentially influential comorbidities or medications were inquired. For muscle echo intensity comparison, 71 healthy controls were prospectively examined. The nerve ultrasound data of the 37 retrospectively included patients have been published previously [18].

### Clinical and Paraclinical Examinations

Muscle strength was clinically evaluated according to the medical research council (MRC) in a range from 0 to 5/5 points. MRC values of the deltoid and biceps brachii muscles, wrist elevation, tibial anterior, iliopsoas, and quadriceps femoris muscle, and toe elevation on both sides were summarized in a score ranging from 0 (total paralysis) to 70 (full muscle strength) points. We additionally assessed a full sensory status and summarized our clinical examination results using the well-established CMT neuropathy score CMTES-2, ranging from 0 to 28 points with higher scores representing a greater disease severity.

### Nerve and Muscle Ultrasound

For the HRUS examination, we used a high-frequency broadband linear array 14 MHz probe, Mindray TE7. The ultrasound examiner was blinded to the patients’ diagnosis.

To assess nerve cross-sectional areas (CSA), we scanned 10 peripheral nerves at 14 landmarks, following the ultrasound pattern sum score (UPSS) protocol, ranging from 0 points (no nerve enlargement) to 22 points (all nerves enlarged) (Suppl. Table 1) [18]. Additionally, we measured CSAs of the median nerve proximal to the carpal tunnel, and of the ulnar nerve at the cubital tunnel. CSA ratios between wrist and forearm for the median nerve and cubital tunnel and upper arm for the ulnar nerve were calculated according to standard entrapment site scanning protocols [19]. To evaluate nerve morphology in its whole extent, we used a modified version of the homogeneity score (HS) (Suppl. Table 1) [20]: nerve segments at non-entrapment sites of the median (UPSS measure points: upper arm, elbow, forearm), ulnar (upper arm, forearm), and tibial (popliteal fossa and ankle) nerve were analyzed, and the pattern of CSA enlargement was scored for each nerve. The HS ranges from − 3 points (focal nerve enlargement in all 3 nerves) to 9 points (all nerve segments are > 150% enlarged) (Suppl. Table 1). We further measured the muscle echo intensity of the tibial anterior muscle (TA), the gastrocnemius muscle including both the medial and lateral heads (GCNM/L), the brachioradial, the dorsal interosseous 1 (IOD1), and the sternocleidomastoid (SCM) muscle, each on the non-dominant side. For semiquantitative classification, we used the grading scale by Heckmatt [21], ranging from 1 (normal muscle) to 4 (maximum altered muscle) points. For an objective analysis, we converted muscle ultrasound images to 8-bit grey value pictures and performed a grey scale histogram analysis with the software ImageJ [22]. Depending on the ultrasound focal zone, a region of interest was marked in order to be able to record as broad a spectrum of echogenic changes in the muscle as possible. For Heckmatt Scoring, the highest echo intensity was evaluated. For comparison with clinical and paraclinical parameters, we summarized all muscle echo intensities (EI sum), using complete muscle data sets only.

### Nerve Conduction Studies

Nerve conduction studies (NCS) were performed in the corresponding nerves using standard neurophysiology devices (Natus Medical Inc. Dantec® Keypoint®, Pleasanton, CA, USA, and Natus Neurology, Nicolet EDX) as previously described. In orientation on the CMTNS-2 score, we measured motor nerve conduction velocities (NCV), distal motor latencies (dmL), and compound motor action potentials (CMAP) of the median and ulnar nerve as well as sensory nerve action potentials (SNAP) and NCVs of the radial nerve at the non-dominant side, respectively. If eligible, NCS of tibial, fibular, and sural nerves were analyzed as well. We categorized nerve conduction patterns using the two well-established classifications by Dyck et al. and Bischoff et al. [4, 23]. The former distinguishes axonal and demyelinating damage patterns by analysis of NCVs of the median or ulnar nerve alone, whereas the latter reflects the clinical routine by evaluation of all available nerves and parameters.

### Statistical Evaluation

The original dataset was implemented into IBM SPSS Statistics version 27 (Chicago, IL, USA) and GraphPad software Inc. GraphPad Prism version 7 (San Diego, CA, USA) and Microsoft Excel 2010 (Redmond, WA, USA). To compare one group with another, we used the Student’s *t*-test for normally distributed and the Mann–Whitney-Wilcoxon test for non-parametric data. Gaussian distribution was tested with the Kolmogorow-Smirnow, D’Agostino and Pearson omnibus, and Shapiro–Wilk normality tests. Group comparisons were performed using one-way ANOVA or the Kruskal–Wallis test if non-parametric. The p-levels were corrected for multiple comparisons with the Tukey–Kramer or Dunn’s post-test method. Linear regression analyses were done to assess clinical, paraclinical, and score correlations.

## Results

### Clinical Data

Between May 2015 and March 2021, we included a total of 150 patients. Demographic data such as height, weight, BMI, and age are summarized in Table 1, together with UPSS, CMTNS-2, and muscle echo intensity values. On the basis of genetic testing and nerve conduction study results, we classified the patients into seven groups: CMT1A, other CMT1 and 4, CMTX, HNPP, CMT2, ATTRv amyloidosis (symptomatic patients and carriers), and Fabry’s disease.

**Table 1:** Demographic data, ultrasound values, and clinical scores

	**Total**	**CMT1A**	**Other CMT1/4**	**CMTX**	**HNPP**	**CMT2**	**ATTRv amyloidosis**		**Fabry’s disease**
No. patients	150	55	28	15	16	15	symptomatic 10	carrier 4	7
Gender female:male	76:74	33:22	12:16	5:10	8:8	8:7	2:8	3:1	5:2
Age (years)	47.88 ± 14.49 (18–79)	48.42 ± 15.37 (18–79)	50.14 ± 12.5 (28–73)	47.99 ± 13.82 (19–69)	41.88 ± 13.54 (21–70)	46.07 ± 14.75 (20–68)	59.8 ± 12.41 (39–79)	48.75 ± 12.45 (31–32)	36.57 ± 11.99 (19–55)
Height (cm)	171.14 ± 10.08 (150–205)	168.8 ± 9.35 (150–188)	172.32 ± 12.05 (150–205)	171.07 ± 5.74 (160–180)	178.4 ± 9.46 (166–194)	173.0 ± 11.46 (160–195)	169.6 ± 8.9 (156–182)	160.5 ± 1.73 (159–163)	173.14 ± 8.71 (163–187)
Weight (kg)	78.55 ± 19.34 (45–143)	75.97 ± 19.67 (45–143)	84.1 ± 23.97 (48–140)	84.33 ± 15.68 (57–120)	83.75 ± 18.05 (65–112)	76.1 ± 18.64 (57–119)	73.25 ± 12.85 (60–95)	65.0 ± 8.72 (59–75)	72.5 ± 10.61 (65–80)
BMI (kg/m^2^)	26.65 ± 5.25 (17–46)	26.78 ± 5.94 (17–43)	27.7 ± 5.8 (19–46)	28.5 ± 5.13 (20–41)	25.88 ± 3.56 (22–33)	24.55 ± 4.32 (17–31)	25.38 ± 3.54 (20–30)	25.33 ± 3.21 (23–29)	25.0 ± 4.24 (22–28)
UPSS (points)	8.36 ± 7.97 (0–22)	15.8 ± 5.86 (1–22)	7.93 ± 7.65 (0–21)	4.07 ± 4.33 (0–17)	1.88 ± 1.99 (0–5)	1.33 ± 1.45 (0–4)	4.5 ± 3.5 (0–10)	1.25 ± 1.89 (0–4)	0.29 ± 0.49 (0–1)
Homogeneity score (points)	2.25 ± 3.89 (− 3–9)	5.7 ± 3.18 (− 1–9)	2.11 ± 3.8 (− 2–9)	− 0.2 ± 2.3 (− 3–6)	− 0.88 ± 0.88 (− 2–0)	− 0.47 ± 0.99 (− 2–2)	− 0.56 ± 2.19 (− 3–3)	− 0.75 ± 1.5 (− 3–0)	− 0.29 ± 0.49 (− 1–0)
Muscle EI (AU)	480.37 ± 79.08 (301–657	497.67 ± 79.51 (361–656)	486.57 ± 78.83 (319–581)	491.14 ± 83.33 (337–613)	471.17 ± 57.91 (395–587)	494.35 ± 55.2 (438–613)	426.94 ± 115.93 (301–571)	Not determined	398.08 ± 50.06 (346–470)
CMTNS-2 (points)	15.38 ± 7.34 (0–33)	18.12 ± 5.25(7–33)	17.37 ± 7.51 (6–30)	20.33 ± 5.74 (10–25)	10.4 ± 6.24 (2–19)	15.11 ± 7.72 (4–26)	11.33 ± 5.43 (3–19)	0.33 ± 0.58 (0–1)	6.71 ± 3.2 (4–12)
CMTES-2 (points)	11.64 ± 5.73 (0–25)	13.02 ± 4.99 (2–25)	12.74 ± 5.84 (3–23)	14.08 ± 5.33 (6–20)	8.5 ± 5.17 (1–16)	13.44 ± 5.53 (6–22)	10.33 ± 4.85 (3–16)	1.25 ± 1.9 (0–4)	6.0 ± 2.51 (4–10)
NCS category	ax: 23 dem: 99 normal: 15 not class: 13	ax:0 dem: 49 normal: 0 not class: 6	ax: 1 dem: 26 normal: 0 not class: 1	ax: 1 dem: 11 normal: 1 not class: 2	ax: 2 dem: 9 normal: 3 not class: 2	ax: 10 dem: 2 normal: 2 not class: 2	ax: 7 dem: 1 normal: 2 not class: 0	ax: 0 dem: 0 normal: 4 not class: 0	ax: 2 dem: 2 normal: 3 not class: 0

Summary of mean values and standard deviations (SD) of demographic data, ultrasound values, and clinical scores. Range is given in brackets. Nerve conduction studies were evaluated using the Bischoff definition

*AU* arbitrary unit, *ax* axonal, *CMTNS-2* Charcot-Marie-Tooth neuropathy score version 2, *CMTES-2* Charcot-Marie-Tooth examination score version 2, *dem* demyelinating, *not class* not classifiable, *EI* echo intensity, *NCS* nerve conduction studies, *UPSS* ultrasound pattern sum score

The most frequent mutation was the CMT1A-associated heterozygous *PMP22* duplication, accounting for 36.7% (*n* = 55). The *PMP22* deletion, clinically presenting as HNPP, was detected in 15 (10%), and a *PMP22* point mutation in three additional patients (one associated with an HNPP phenotype, two with a CMT phenotype (CMT1E)). *GJB1* mutations (CMTX1) were found in 10 male and 5 female patients (*n* = 15, 10%). *MPZ* mutations were present in 16 cases (10.7%) and were assigned to CMT1B (*n* = 14) or CMT2J (*n* = 2), according to nerve conduction studies and the clinical presentation. In addition, we included 14 individuals (9.3%) with known amyloidogenic *TTR* mutations, of whom 10 were stage 1 ATTRv amyloidosis patients and 4 were asymptomatic carriers. The Fabry’s disease cohort, caused by *GLA* mutations, consisted of 2 male and 5 female patients (*n* = 7, 4.7%). All genes and variants are summarized in Suppl. Table 2.

#### Demographic Data

Age was significantly different in patients with ATTRv amyloidosis and Fabry’s disease (mean 56.64 vs 37.57 years; *p* = 0.04). In all other groups, no significant difference was demonstrated, and weight and BMI showed no significant difference among all subgroups (Table 1). Except for one Asian patient, all patients were of Caucasian origin. The muscle group controls were slightly younger than the patients whose muscles were analyzed (age mean control = 45.04 ± 16.94 years vs mean muscle patients = 50.16 ± 13.83, *p* = 0.04). No difference in height, weight, and BMI was observed.

#### CMTNS-2, CMTES-2, and MRC

The CMT1A and CMTX cohort had the highest CMTNS-2 scores in comparison to HNPP, symptomatic ATTRv amyloidosis, and Fabry’s disease patients (Fig. 2A, mean values Table 1; *CMT1A* vs HNPP, *p* = 0.007, vs sym. ATTRv, *p* = 0.04, vs Fabry’s disease, *p* = 0.0002; *CMTX* vs HNPP, *p* = 0.008, vs sym. ATTRv, *p* = 0.03, vs Fabry’s disease, *p* = 0.0003; CMT1/4 vs Fabry’s disease, *p* = 0.002). The CMTES-2 comparison revealed similar results: patients in the symptomatic ATTRv amyloidosis and Fabry’s disease cohort, but not HNPP patients, showed significantly lower scores in comparison to the clinically most impaired patients in the CMT1A and CMTX groups (mean values are enlisted in Table 1; sym. ATTRv vs CMT1A, *p* = 0.023, vs CMTX, *p* = 0.038; Fabry’s disease vs CMT1A *p* = 0.024, vs CMTX, *p* = 0.026). In accordance with these findings, the MRC sum was highest in the Fabry’s cohort in comparison to all other cohorts except for HNPP and symptomatic ATTRv patients (Fabry’s disease MRC sum mean = 69.43 ± 0.98 vs CMT1A = 61.52 ± 7.2, *p* = 0.003, vs CMT1/4 = 62.62 ± 7.3, *p* = 0.17, vs CMTX = 59.0 ± 7.5, *p* = 0.002, vs HNPP = 65.5 ± 6.0 *p* = 0.7, vs CMT2 = 58.55 ± 1 0.8, *p* = 0.006, vs sym. ATTRv = 63.5 ± 8.1, *p* = 0.07).

### Nerve Ultrasound Findings

#### CMT1A

**Fig. 1:**
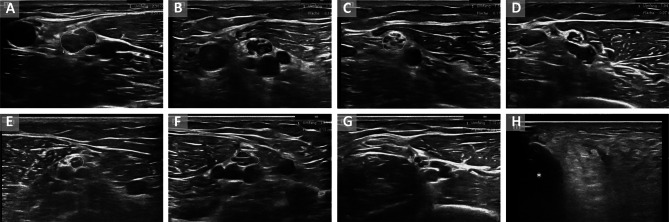
Ultrasound examples. A-G cross-sectional areas (CSA) of median nerves at the upper arm in patients with different hereditary polyneuropathies,** A** CMT1A, 33 m^2^, **B** other CMT1/4 (in this example CMT1B), 19 mm^2^, **C** CMTX, 17 mm^2^, **D** HNPP, 7 mm^2^, **E** CMT2F, 8 mm^2^, **F** ATTRv amyloidosis stage 1, 15 mm^2^, **G** Fabry’s disease, 8 mm^2^, **H** example of muscle alteration of tibial anterior muscle in a patient with CMT 1B (variant in *MPZ*), asterix: tibia

**Fig. 2:**
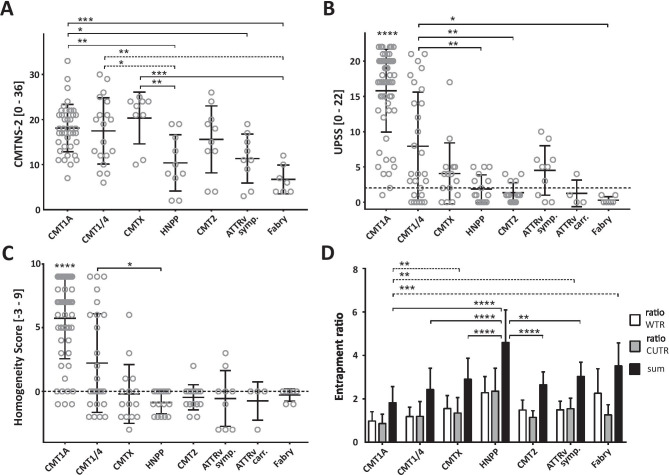
Scatter Plot of CMTNS-2, UPSS, Homogeneity Score and bar graph of entrapment ratio. **A.** Presentation of CMTNS-2 scores. Patients with CMT1A achieved the highest values, patients with HNPP the lowest. **B** Patients with CMT1 or 4 exhibited the highest UPSS in comparison to all other neuropathy groups (CMT1A UPSS mean = 15.8 ± 5.86, p = 0.0001). **C** Nerve enlargement in CMT1A patients was detected all along the nerve axis (Homogeneity Score (HS) mean = 5.7 ± 3.18), whereas focal nerve enlargements were detectable in symptomatic TTR amyloidosis patients (HS mean = -0.56 ± 2.19). *In B and C, dotted lines represent the upper normal value cut off: UPSS 2 points, Homogeneity Score 0 points.* **D.** entrapment ratio of wrist to forearm, cubital tunnel to upper arm, and the sum of both. The highest ratios were seen in HNPP patients (ratio sum mean = 4.63 ± 1.45; p < 0.0001). ***Abbreviations:*** CMTNS-2 = Charcot-Marie-Tooth neuropathy score version 2; CUTR = cubital tunnel to upper arm ratio; UPSS = Ultrasound pattern sum score; WTR = wrist to forearm ratio

The CMT1A cohort showed the highest nerve enlargement in comparison to all other subgroups (Table 1; Fig. 1A, 2B; UPSS mean = 15.8 ± 5.86, *p* = 0.0001). An UPS score ≥ 8.5 points discriminated best between CMT1A and all other neuropathies, a score ≥ 9.5 between CMT1A and other demyelinating neuropathies (ROC curve analysis AUC = 0.91 sensitivity = 0.84, specificity = 0.84 if UPSS ≥ 8.5 or AUC = 0.86 sensitivity = 0.8, specificity = 0.74 if UPSS ≥ 9.5).

If nerve enlargement was seen, all nerves were enlarged in the entire course without any predilection site, in contrast to other demyelinating, immune-mediated neuropathies. Therefore, the homogeneity score revealed the highest points in comparison to all other subgroups (mean = 5.7 ± 3.18, *p* = 0.0001; Fig. 2C; Table 1), reflecting the generalized nerve enlargement (AUC = 0.88 among all groups, sensitivity = 0.82, specificity = 0.86 for HS ≥ 2.5). The wrist-to-forearm ratio (WTR) for the median nerve and cubital tunnel-to-upper arm ratio (CUTR) for ulnar nerve was significantly lower in comparison to most of the other subgroups, suggesting no additional nerve enlargement at entrapment sites (mean CMT1A WTR + CUTR = 1.84 ± 0.78, vs mean CMTX = 2.9 ± 0.97, *p* = 0.01, vs mean HNPP = 4.63 ± 1.45, *p* < 0.0001, vs mean Fabry’s disease = 3.34 ± 1.08, *p* < 0.0001; Fig. 2D).

#### CMT1/4

In comparison to CMT1A patients, the second primarily demyelinating subgroup, other CMT1 and CMT4, had a lower UPSS, but still significantly enlarged CSAs in comparison to the axonal subgroups (UPSS mean values are enlisted in Table 1; example is given in Fig. 1B; CMT1/4 vs HNPP *p* = 0.006, vs CMT2 *p* = 0.003, vs Fabry’s disease *p* = 0.013; Fig. 2B). Nerve enlargement was less homogenous than in the CMT1A group and differed significantly from the focally restricted HNPP patients (HS mean values; Table 1, vs HNPP *p* = 0.013; Fig. 2C).

The main characteristic ultrasound findings in ***HNPP*** patients were a high WTR (median nerve) and CUTR (ulnar nerve) (mean WTR + CUTR = 4.63 ± 1.45; *p* < 0.0001 in comparison to all groups except Fabry’s disease (Fig. 2C); AUC 0.94, sensitivity = 0.88 and specificity = 0.9 for HS ≥ 3.56) and a rather low UPSS, showing mostly normal nerve segments in between the entrapment sites (mean UPSS = 1.88 ± 1.99; Figs. 1D and 2B).

In symptomatic ***ATTRv*** patients, the UPSS ranged on a comparable level with ***CMTX*** (mean = 4.5 ± 3.5 vs 4.07 ± 4.33; Figs. 1F and 2B), and nerve enlargement was focally pronounced (HS =  − 0.56 ± 2.19; Fig. 2C). Recognizing that these case numbers were too low to be representative for statistical analyses, the UPSS still tended to be higher in symptomatic patients compared to pre-symptomatic *TTR* mutation carriers and to other axonal neuropathy patients (CMT2, mean = 1.33 ± 1.45). In symptomatic ATTRv amyloidosis patients, the cervical nerve roots five and six appeared to be enlarged, which was not the case in peripheral sensory nerves.

### Nerve Conduction Studies

Using the default classification system for hereditary neuropathies by Dyck et al., nerve conduction studies (NCS) revealed a primarily axonal damage pattern in 16 patients, a demyelinating one in 71, an intermediate one in 15, and normal arm NCS in 34 patients. Due to unobtainable nerve action potentials, 14 NCS could not be classified.

We further applied another standard classification system for polyneuropathies by Bischoff et al., typically used to categorize all types of neuropathies in clinical routines [23]: 23 patients had an axonal alteration of NCS, 99 demyelinating NCS, 15 normal, and 13 unclassifiable NCS (Table 1).

In the following, we will use the classification system by Bischoff.

### Correlations of Clinical Parameters, Nerve Conduction Studies, and Ultrasound Parameters

**Fig. 3:**
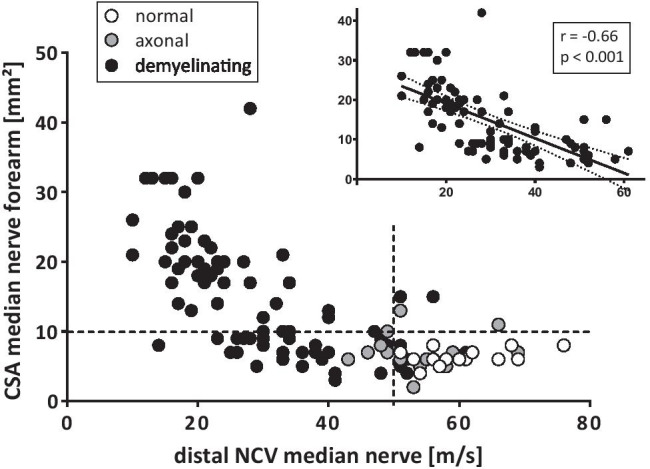
Nerve conduction velocity in comparison to cross-sectional area of the correlating nerve segment. Black dots: all polyneuropathies with demyelinating nerve conduction studies, grey: primarily axonal damage pattern and white normal measurements. In demyelinating neuropathies, nerve conduction velocity and cross-sectional area of the respective nerve segment correlated inversely (r = -0.66, R^2^ = 0.44, p < 0.001, inlet). *The dotted lines indicate the upper limit cut-off value: CSA median nerve 10 mm*^*2*^*, NCV according to Dyck* = *45 m/s. ****Abbreviations:*** CSA = cross-sectional area; NCV = nerve conduction velocity

The NCV correlated inversely with the CSA of the corresponding nerve segment in demyelinating neuropathies (Fig. 3; CSA median nerve forearm vs distal NCV *r* =  − 0.66; *R*^2^ = 0.43, *p* < 0.001; CSA ulnar nerve forearm vs distal NCV *r* =  − 0.73, *R*^2^ = 0.53; *p* < 0.001). In axonal neuropathies, NCS did not correlate with CSA values.

**Fig. 4:**
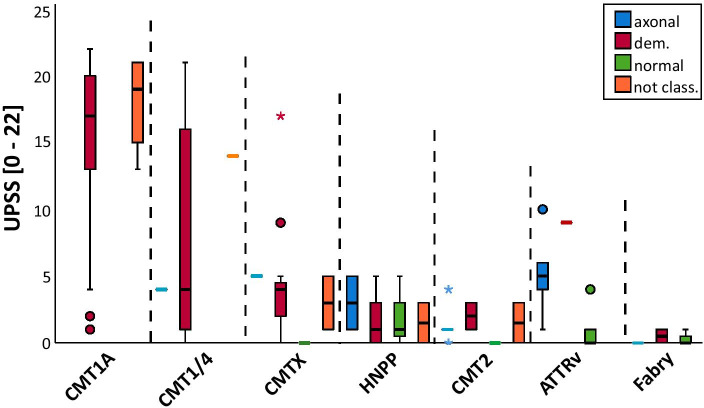
Comparison of nerve conduction studies and UPSS. UPSS was significantly higher in demyelinating polyneuropathies in comparison to axonal polyneuropathies or polyneuropathies with normal nerve conduction studies (NCS). In 14 patients, nerve ultrasound allowed further classification into different polyneuropathy groups, whereas NCS were not classifiable due to abolished nerve signals. On the other hand, in nine CMT1A patients, nerve ultrasound was only slightly altered with typical signs of demyelination in the NCS (distal median nerve mean NCV = 27 m/s).* Abbreviations*: UPSS = Ultrasound pattern sum score; not class. = not classifiable

In demyelinating neuropathies, the UPSS differed significantly between neuropathies with axonal damage and normal NCS (mean UPSS demyelinating = 10.44 ± 8.06, UPSS axonal = 2.91 ± 2.7, UPSS normal = 0.8 ± 1.57, *p* < 0.0001; Fig. 4). In HNPP, ATTRv, and Fabry’s patients, NCS results were heterogenous, whereas the UPSS was in the same range in each neuropathy group. In nine CMT1A patients (16.3%), the ultrasound revealed only moderately enlarged nerves (UPSS mean = 4.44 ± 1.83; Fig. 4), whereas NCS showed typical signs of a demyelinating neuropathy with slow NCVs (distal median nerve mean NCV = 27 m/s).

UPSS correlated with clinical scores CMTNS-2 (*r* = 0.4, *p* < 0.001), while cMAP did not correlate with MRC sum or CMTES-2.

### Muscle Echo Intensity

**Fig. 5:**
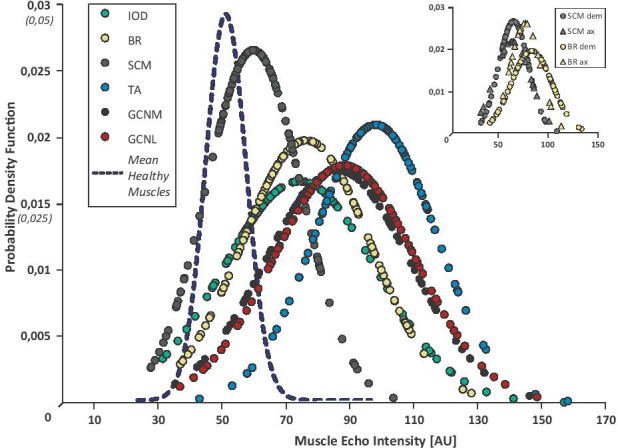
Muscle echo intensities of different muscles. In all examined polyneuropathies, the tibial muscle was most commonly altered resulting in higher echo intensities (EI) (EI TA mean = 98.24 ± 17.52). EI of SCM, BR/IOD, GCNM/L and TA differed significantly, but no difference was detected between the axonal and demyelinating groups (inlet). Mean EI values were higher in all muscles in comparison to unaffected muscles of healthy control persons (p < 0.0001). *The mean of all control muscles is shown as dotted line, the scaling of y-axis is indicated in italic letters. Abbreviations:* ax = axonal; BR = brachioradialis muscle; dem = demyelinating; IOD: interosseous muscle one; GCNM/L = lateral/ medial head of gastrocnemius muscle; SCM = sternocleidomastoid muscle; TA = tibialis anterior muscle

We analyzed the muscle echo intensity (EI) in 651 muscles, examined in 124 patients, and 417 muscles of 71 healthy controls. Most commonly, we found EI changes in the TA muscle reflecting the length-dependency of the axonopathy: With the highest overall EI (mean = 98.24 ± 17.52; Fig. 5), 88 of 102 TA muscles (86.3%) reached Heckmatt scores > 1. We found inverse correlations between TA EI and dorsal foot extension strength (*r* =  − 0.39, *p* < 0.0001), between interosseous dorsalis (IOD1) EI and finger spreading (*r* =  − 0.56, *p* < 0.001), and between the EI of both heads of the gastrocnemius muscles (GCNL/M) and foot flexion (GCNM *r* =  − 0.33, *p* = 0.001, GCNL *r* =  − 0.3, *p* = 0.003). As indicated in Fig. 5, the sternocleidomastoid muscle (SCM) had the lowest EI values (mean = 60.08 ± 15.02), suggesting a rather late or lower clinical manifestation of cranial and proximal spinal nerves, which correlated with the clinical examination. We found similar EIs comparing the lateral with the medial head of the GCN muscles, and neither deviated EI values between the brachioradialis (BR) and IOD1 muscles. In contrast, the EIs of the SCM, BR/IOD, GCNM/L, and TA differed significantly compared to each other (EI sum mean ± SD: BR = 76.39 ± 20.57; SCM = 60.08 ± 15.02; IOD1 = 75.1 ± 25.07; GCNM = 86.53 ± 23.06; GCNL = 88.54 ± 22.75; *p* < 0.0001; Fig. 5). EI values of all patient muscles were significantly higher than in control muscles (*p* < 0.0001 for all muscles, mean EI healthy muscles IOD = 40.84 ± 23.85; BR = 41.59 ± 14.73; SCM = 48.67 ± 12.98; TA = 59.24 ± 18.14; GCNM = 52.27 ± 16.62; GCNL = 55.05 ± 18.3). No significant EI difference was seen between the neuropathy groups or comparing axonal and demyelinating neuropathies (Fig. 5, small part).

**Fig. 6:**
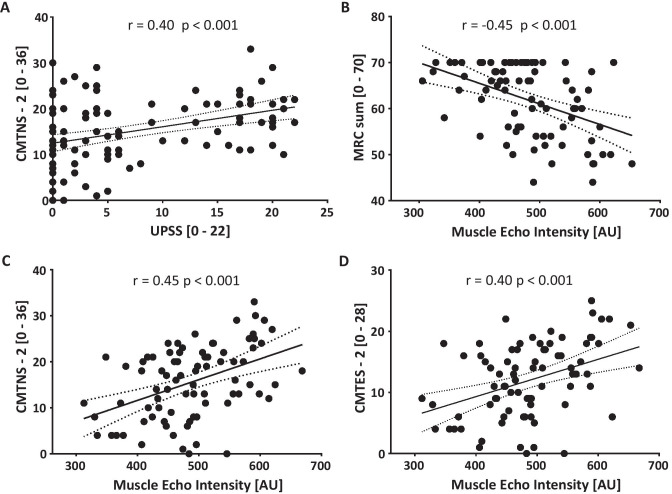
Correlation of nerve and muscle ultrasound parameters with clinical scores. **A**. The CMTNS-2 correlated with the UPSS (r = 0.4, R^2^ = 0.16 p < 0.0001). **B-D.** For further analyses, the sum of muscle echo intensities (EI sum) was calculated, and only complete data sets of muscle values were used. CMTNS-2 and CMTES-2 showed a positive correlation and the MRC sum an inverse correlation with EI sum (*EI sum vs MRC sum* r = -0.45, R^2^ = 0.2, p < 0.0001; vs *CMTNS-2* r = 0.45, R^2^ = 0.2, p < 0.0001; vs *CMTES-2* r = 0.4, R^2^ = 0.16, p < 0.0001). *Abbreviations:* CMTES-2 = Charcot-Marie-Tooth examination score version 2; CTMNS-2 = Charcot-Marie-Tooth neuropathy score version 2. MRC = Medical Research Council’s scale of muscle power; UPSS = Ultrasound pattern sum score

Regarding clinical parameters, the patients’ age and estimated disease duration correlated with the EI sum (age *r* = 0.46, *p* < 0.001; estimated disease duration *r* = 0.35, *p* = 0.0007). The CMTNS-2 and CMTES-2 both correlated significantly, and the MRC sum score showed an inverse correlation with the EI sum (Fig. 6B–D; CMTNS-2, *p* < 0.001; CMTES-2, *p* < 0.001; MRC sum, *p* < 0.001).

## Discussion

Hereditary neuropathies are heterogeneous in etiologies and clinical patterns. This study aims at characterizing and differentiating hereditary neuropathies by muscle and nerve ultrasound, a non-invasive, radiation-free, dynamic, and repeatable method that is convenient for both patients and examiners. By combining UPSS, homogeneity score, and entrapment ratios, the nerve ultrasound depicted characteristic features of nerve alteration for each hereditary neuropathy. In accordance with previous studies [18], the UPSS was highest in CMT1A patients, followed by other demyelinating CMT forms, whereas it was lowest in axonal CMT and Fabry’s disease. Diseases like HNPP, CMTX, and ATTRv amyloidosis all placed in the middle rows of our ranking with certain nerve morphology patterns. This reflects the partial, but not continuous demyelination that has been attributed to liability to pressure in HNPP, to the intermediate character of neuropathy in CMTX, and the partial Schwann cell involvement in ATTRv amyloidosis [24, 25]. Between the different cohorts, patients with CMT1A had the highest homogeneity score, representing continuous nerve enlargement. The highest entrapment ratios were observed in HNPP patients, who, besides the typical entrapment sites, had low UPSS values. Other diseases prone to entrapment were stage 1 ATTRv amyloidosis and Fabry’s disease. ATTRv amyloidosis patients also featured regional and proximal dominant nerve enlargement, which tended to increase by disease progression. This finding has significant impact with regard to inflammatory neuropathies such as chronic inflammatory demyelinating polyneuropathy.

In accordance with the literature [26], we found an inverse correlation between NCV and the CSA, reflecting nerve thickness in demyelinating neuropathies. The clinical and NCS-based CMTNS-2 score correlated with the UPSS as well. Comparisons of NCS and HRUS revealed the strengths and weaknesses of each method: whereas NAPs were no longer measurable in 14 patients, the HRUS showed typical nerve alterations for each neuropathy. Additionally, NCS patterns were heterogeneous suggesting both signs of axonal and demyelinating nerve damage in HNPP, ATTRv, and even CMT2 patients; HRUS ranged in the same UPSS levels in these subgroups, thus enabling a more precise diagnosis. In nine CMT1A patients, however, UPSS values were unexpectedly low despite typically slow NCVs.

NCS are a good method to measure cMAP amplitudes in distal muscles. When assessing the involvement of more proximal muscles, nerve and muscle ultrasound is the more appropriate method. Another factor is that ultrasound examinations are much better tolerated, especially for follow-up examinations, than NCS examinations.

To increase sensitivity and specificity, the authors suggest combining both methods.

In the overall cohort, the muscle echogenicity correlated inversely with the respective muscle’s strength not only in the echogenicity sum, but when referring to individual muscles as well. This was independent from the genetic cause and neurophysiological pattern of neuropathy, meaning that muscle ultrasound was a good marker of motor disease severity, but not specific for the underlying type of neuropathy.

We conclude that the nerve ultrasound evolves its highest potential for differential diagnostic considerations when used for pattern analysis including the UPSS, the homogeneity, and entrapment score. Reflecting secondary degeneration of muscle and its transformation into connective tissue, the muscle sonography, a novel method in the context of hereditary neuropathies, might serve as a marker for disease duration and course. In future studies, its role as prognostic marker remains to be further elucidated.

As a potential biomarker for CMT1A, muscle MRI fat fraction has been previously discussed showing strong correlations with clinical scores such as the CMTES-2 [27–29]. Despite this promising perspective, sample sizes were low (*n* = 14–20) in the respective studies, and CMT1A patients were not compared to other types of (hereditary) neuropathies. As an additional parameter, muscle diameter and volume were examined in several smaller studies. In an analysis including 114 patients with neuropathies and myopathies, the muscle diameter showed a correlation with clinical and electrophysiological data [30]. In addition to echogenicity, this could represent another biomarker for axonal damage. However, to what extent this parameter is suitable for follow-up is still unclear and needs further prospective investigation. Compared to MRI volumetry, nerve ultrasound is faster and easier in conduction and more cost-efficient. On the other hand, muscle echogenicity does not change homogeneously in polyneuropathies, and atrophic, (still) normal, and re-innervated muscle fibers can present as a “mixed” echogenicity, which makes it more difficult to evaluate by ultrasound. Head-to-head studies of MRI- and ultrasound-derived parameters will be interesting to investigate in the future.

Considering the rareness of the different neuropathy entities, the sample size of 150 is an important strength of this study. As a potential weakness, demyelinating CMT forms and especially CMT1A were relatively overrepresented, which reflects, however, the real-life distributions at clinical centers. As in clinical practice, axonal CMT forms were more heterogeneous being associated with various genes and mutations and therefore involving several pathomechanisms, which might be considered another potential, but inevitable confounder.

Following the great advances of pathomechanism-based treatment in ATTRv amyloidosis, other neuropathies of genetic cause will most likely become treatable in the near future. More than ever, these exciting developments require reliable markers for the earliest possible clinical diagnosis as well as for disease severity and progression.

We conclude that the evaluation of nerve ultrasound patterns is helpful to distinguish different hereditary neuropathy subtypes and that muscle echogenicity might enable an estimation of disease duration and course. Therefore, both nerve and muscle ultrasound might constitute potential parameters of interest in clinical trials, the former to establish patient eligibility and the latter to assess outcome. As further future perspectives, the comparison of diagnostic patterns might help to distinguish between hereditary and acquired neuropathies. Immune-mediated entities such as chronic inflammatory demyelinating polyradiculoneuropathy have, for instance, a more proximal localization and regional distribution of nerve damage and CSA enlargement [31].

For clinical practice, we recommend incorporating muscle and nerve ultrasound into the standard procedures as an add-on tool for diagnosis and follow-up of hereditary neuropathies.

## Supplementary Information

Below is the link to the electronic supplementary material.

Supplementary file1 (PDF 1087 KB)

Supplementary file2 (PDF 1308 KB)

Supplementary file3 (PDF 298 KB)

Supplementary file4 (PDF 1225 KB)

Supplementary file5 (DOCX 21 KB)

Supplementary file6 (PDF 97 KB)

Supplementary file7 (PDF 509 KB)
