# Supplementary material for: New Keys to Early Diagnosis: Muscle Echogenicity, Nerve Ultrasound Patterns, Electrodiagnostic, and Clinical Parameters in 150 Patients with Hereditary Polyneuropathies
Source: Neurotherapeutics. 2021 Oct 27;18(4):2425–35. doi: 10.1007/s13311-021-01141-3 (PMC8804010; doi:10.1007/s13311-021-01141-3)
Supplement: Supplementary file 5 — Supplementary file5 (DOCX 21 KB) [file 13311_2021_1141_MOESM5_ESM.docx]

**Suppl. Table 1.** Ultrasound pattern sum score (UPSS) and Homogeneity Score. Measure points and cut-off values for scoring

| **A. Peripheral sensorimotor nerves (range 0-16 points)** | | | |
| --- | --- | --- | --- |
| Median nerve  *upper arm* | <12 mm² 0 points  ≥12 mm² 1 point  >18 mm² 2 points | Ulnar nerve  *upper arm* | <9.5mm² 0 points  ≥9.5mm² 1 point  >14,25mm² 2 points |
| Median nerve  *elbow* | <12 mm² 0 points  ≥ 12 mm² 1 point  >18 mm² 2 points | Ulnar nerve  *forearm* | <8.5 mm² 0 points  ≥8.5 mm² 1 point  >12.75mm² 2 points |
| Median nerve  *forearm* | <10mm² 0 points  ≥10mm² 1 point  >15mm² 2 points | Peroneal nerve  *popliteal fossa* | <11.5mm² 0 points  ≥11.5mm² 1 point  >17.25mm² 2 points |
| Tibial nerve  *popliteal fossa* | <33mm² 0 points  ≥33mm² 1 point  > 49.5mm² 2 points | Tibial nerve  *ankle* | <14mm² 0 points  ≥14mm² 1 point  > 21mm² 2 points |
| **B. Cervical roots and vagus nerve (range 0-3 points)** | | | |
| Cervical root 5  (diameter) | <2.9mm 0 points  >2.9mm 1 point | Cervical root 6  (diameter) | <4.2mm 0 points  >4.2mm 1 point |
| Vagus nerve  *Carotid segment* | <3.5mm² 0 points  >3.5mm² 1 point |  |  |
| **C. Sensory nerves (range 0-3 points)** | | | |
| Superficial radial nerve  *Arcarde of Frohse* | <3.0mm² 0 points  >3.0mm² 1 point | Superf. peroneal nerve  *Mid lower leg* | <3.5mm² 0 points  >3.5mm² 1 point |
| Sural nerve  *Mid-calf* | <3.5mm² 0 points  >3.5mm² 1 point |  |  |
| **UPSS total range 0 – 22 points** | | | |
| **Homogeneity score (range -3 – 9 points)** | | | |
| Median nerve  *Segments: upper arm, elbow, forearm* | - **-1 pt.:** 1(-2) segment(s) ≤100% *and* 1(-2) segment(s) >100% - **0 pts.:** All segments ≤ 100% - **1 pt.:** segment(s) >100% next to segment(s) >150% - **2 pts.:** All segments >100% but <150% - **3 pts.:** All segments > 150% | Ulnar nerve  *Segments: upper arm, forearm* | - **-1 pt.:** 1 segment ≤100% *and* 1 segment >100% - **0 pts.:** All segments ≤ 100% - **1 pt.:** 1 segment >100% *and* 1 segment >150% - **2 pts.:** All segments >100% but <150% - **3 pts.:** All segments > 150% |
| Tibial nerve  *Segments: popliteal fossa, ankle* | - **-1 pt.:** 1 segment ≤100% *and* 1 segment >100% - **0 pts.:** All segments ≤ 100% - **1 pt.:** 1 segment >100% *and* 1 segment >150% - **2 pts.:** All segments >100% but <150%   **3 pts.:** All segments > 150% |  |  |

**Supplemental table 2. List of genes and variants**

| **CMT1A** | **Other CMT1/4** | **CMT2** | **CMTX1** | **HNPP** | **ATTRv amyloidosis** | **Fabry’s disease** |
| --- | --- | --- | --- | --- | --- | --- |
| ***PMP22* duplication (het) = 55** | ***FIG4* (hom) = 2**  p.Ile41Thr (hom) = 1  del exon 21 (hom) = 1  ***LITAF* = 3**  p.Cys127Tyr (het) = 1  p.Val144Met (het) = 2  ***MPZ* = 14**  p.Lys35fsX66 (het) = 1  p.Arg67Cys (het) = 1  p.Tyr82His (het) = 2  p.Phe95Ser (het) = 1  p.Arg98His (het) = 1  p.Tyr119Cys (het) = 2  p.Thr124Met (het) = 1  p.Gly163Arg (het) = 2  ..p.Gly206* (het) = 2  p.Asp.224Thyr (het) = 1  ***NEFL* = 3**  p.Leu329Pro (het) = 2  p.Gln332Pro (het) = 1  ***PMP22* frame shift mutations = 2**    p.Leu145Argfs*10 (het) = 1    p.Val17Alafs22 (het) = 1  ***SH3TC2* = 4**  p.Arg529Gln and  p.Leu1048Pro (comp. het.) = 1  p.Glu632Lysfs*13 (hom.) = 1  p.Arg954* (het) = 1  p.Arg954* and p.Asn881Ser (comp. het.) = 1 | ***AARS* = 1**  p.His605Gln (het) = 1  ***HSPB1* = 5**  p.Pro39Leu (het) = 1  p.Try42* (het) = 1  p.Ser126Gly (het) = 1  p.ser135Tyr (het) = 1  p.Arg136Leu (het) = 1  ***KIF5A* = 1**  p.Leu262Pro (het)  ***LRSAM1* = 2**  ..p.Glu682_Ala683ins21 = 2  ***MFN2* (het) = 2**  p.Arg94Gln = 1  p.Arg364Trp = 1  ***MME* = 1**  p.Pro156Leufs*14 (het)  ***MORC2* = 1**  p.Arg252Trp (het)  ***MPZ* = 2**  p.Tyr119Cys (het) = 1  p.Thr124Met (het) = 1 | ***GJB1* (het) = 5**  p.Arg75Gln = 1  p.Glu102Argfs = 2  p.Tyr160Cys = 1  p.Thr185Serfs = 1  ***GJB1* (hem) = 10**  p.Val35Ala = 2  p.Ala39Val = 1  p.Arg75Gln = 1  p.Leu81Pro = 1  p.Glu102Argfs = 1  p.Tyr160Cys = 1  p.Pro172Ser = 1  p.Ala207Glyfs = 1  p.Arg220* = 1  **total = 15** | ***PMP22* deletion = 15**  ***PMP22* point mutation = 1**  c.78+5G>C; p.? (het.) = 1 | ***TTR* = 14**  p.Val50Met (het) = 9  p.Leu58His (het) = 1  p.Ala59Aps (het) = 1  p.Glu109Gln (het) = 1  p.Ile127Val (het) = 2 | ***GLA* (hem) = 2**  p.Asp313Tyr = 2  ***GLA* (het) = 5**  p.Ala143Thr = 1  p.Asp313Tyr = 4  **total = 7** |

**Supplemental table 2:** Number of patients per genes and variants are enlisted. Assignment to subgroups was done according to nerve conduction studies, affected genes and clinical presentation. ***Abbreviations:*** **comp. het**.: compound heterozygous; **hem:** hemizygous, **het:** heterozygous; **hom:** homozygous
